# Variability in Nutrient Use by Orchid Mycorrhizal Fungi in Two Medium Types

**DOI:** 10.3390/jof9010088

**Published:** 2023-01-06

**Authors:** Alžběta Novotná, Sophie Mennicken, Caio C. Pires de Paula, Hélène Vogt-Schilb, Milan Kotilínek, Tamara Těšitelová, Petr Šmilauer, Jana Jersáková

**Affiliations:** 1 Department of Ecosystem Biology, Faculty of Science, University of South Bohemia, Branišovská 1760, 37005 České Budějovice, Czech Republic; 2 Institute of Hydrobiology, Biology Centre CAS, Na Sádkách 702/7, 37005 České Budějovice, Czech Republic; 3 Centre d’Écologie Fonctionnelle et Évolutive, Centre National de la Recherche Scientifique, University of Montpellier, EPHE, IRD, 1919 Route de Mende, 34293 Montpellier, France

**Keywords:** nutrient utilization, Orchidaceae, rhizoctonia, solid medium, liquid medium, Tulasnellaceae, Ceratobasidiaceae, Serendipitaceae

## Abstract

Orchid mycorrhizal fungi (OMF) from the rhizoctonia aggregate are generally considered to be soil saprotrophs, but their ability to utilize various nutrient sources has been studied in a limited number of isolates cultivated predominantly in liquid media, although rhizoctonia typically grow on the surface of solid substrates. Nine isolates representing the key OMF families (Ceratobasidiaceae, Tulasnellaceae and Serendipitaceae), sampled in Southern France and the Czech Republic, were tested for their ability to utilize carbon (C), nitrogen (N) and phosphorus (P) sources *in vitro* in both liquid and solid media. The isolates showed significant inter- and intra-familiar variability in nutrient utilization, most notably in N sources. Isolates produced generally larger amounts of dry biomass on solid medium than in liquid one, but some isolates showed no or limited biomass production on solid medium with particular nutrient sources. The largest amount of biomass was produced by isolates from the family Ceratobasidiaceae on most sources in both medium types. The biomass production of Tulasnellaceae isolates was affected by their phylogenetic relatedness on all sources and medium types. The ability of isolates to utilize particular nutrients in a liquid medium but not a solid one should be considered when optimizing solid media for symbiotic orchid seed germination and in understanding of OMF functional traits under *in situ* conditions.

## 1. Introduction

Most terrestrial plants form close relationships with mycorrhizal fungi to improve access to soil nutrients [1]. Different fungal families can, however, utilize various nutrient sources [2], which may help the associated plants to colonise different habitat niches. The orchid family is obligately dependent on mycorrhizal symbiosis from early ontogenetic stages because germinating seeds lack nutrient reserves, thus fully depending on carbon (C), nitrogen (N) and phosphorus (P) provided by orchid mycorrhizal fungi (OMF) [1,3]. These fungi mostly belong to the basidiomycetous families; Ceratobasidiaceae and Tulasnellaceae from Cantharellales, and Serendipitaceae from Sebacinales of the former rhizoctonia aggregate [4]. Besides forming endomycorrhizal associations with orchids, rhizoctonian fungi can occupy various ecological niches [5]. They have been detected as endophytes of other plant families [6,7], as ectomycorrhizal fungi on tree roots [7,8] or as plant pathogens [9,10]. Their predominant nutritional mode is, however, saprotrophy via the decomposition of dead organic matter in the soil [2,11,12].

In terrestrial orchid habitats, the highest abundance of OMF is found in the first 5 cm of the soil layer [13], which correlates with the high occurrence of dead organic matter at a depth of 4–12 cm [14]. Dead organic matter consists of plant and animal residues in different stages of decomposition and is thus rich in various forms of C, N and P, together with vitamins and other minerals. As with other Basidiomycetes, OMF form a mycelial net, secreting hydrolytic enzymes essential for the digestion of dead organic matter [2,15]. Plant polysaccharides are the most abundant C sources for soil micro-organisms and can be divided into plant cell wall polysaccharides (cellulose, hemicellulose and pectin), complex polyphenolic polymers (lignin) and storage polysaccharides (starch, inulin or trehalose) [16]. Soil micro-organisms decompose dead organic material via numerous carbohydrate-active enzymes (CAZymes) and subsequently utilize the available C to synthetize their own biomass or provide the accessible C forms (e.g., nucleic acids) to other organisms [2,17]. Nitrogen and P compounds occur in soil in considerably lower quantities compared with C, and mycorrhizal fungi can use various N and P forms which would otherwise remain largely unavailable to plant roots. More than 90% of the soil N consists of organic amino acids and amino sugars, which need to be mineralized by micro-organisms into inorganic NH_4_^+^ and NO_3_^−^, which are readily taken up by plants [18]. Soil P exists predominantly in immobile inorganic fractions, which are either adsorbed to soil mineral surfaces or occur as sparsely available precipitates [19]. In its organic form, P is found in inositol phosphates, phosphonates, orthophosphate monoesters and organic polyphosphates. Both P forms are microbially transformed via lytic enzymes into more easily accessible inorganic salts of orthophosphoric acid (H_2_PO_4_^−^, HPO_4_^2−^) [20].

Available in vitro studies investigating nutrient uptake by OMF (Ceratobasidiaceae, Tulasnellaceae, Serendipitaceae strains) cultivated on various C-, N- and P-enriched substrates [21,22,23,24] have been performed predominantly with strains isolated from numerous Australian terrestrial orchids (but see [25] investigating an Italian *Tulasnella calospora* strain). Although it is difficult to generalise the results of these studies, which seem contradictory in some cases, OMF usually form a larger biomass on media with complex carbohydrates, amino acids and inorganic orthophosphates. The fungi have also showed inter-familiar [22], interspecific [23] and even intraspecific variability [24,26] in resource utilization. For example, Tulasnellaceae isolates do not utilize nitrates, whereas those from the family Ceratobasidiaceae can effectively use nitrate [22]. All three rhizoctonian families are, however, genetically highly diverse [4,27,28,29], and investigations of nutrient utilization abilities in phylogenetically distant fungal strains collected outside Australia may have ecological and conservation implications, e.g., for optimisation of *in vitro* symbiotic propagation media [26].

Although the majority of filamentous Basidiomycetes are strictly aerobic and grow at the surface or inside solid substrates in their natural habitats, most of the experimental data on the ability of OMF to metabolise C, N and P have been obtained by growing strains in a liquid culture (see the above-mentioned studies). Yet, the physiology and developmental biology of fungi strongly differ depending on whether they grow on a solid substrate or submerged in liquid media [30,31]. For example, some fungal isolates are good producers of enzymes on solid but not in liquid media [32]. In addition, some filamentous fungi cannot even grow in submerged cultures [30] or require the addition of vitamins (such as thiamine and *para*-amino benzoic acid (PABA)) for growth in liquid conditions, such as strains from the Tulasnellaceae family [22]. Therefore, the cultivation of OMF solely in liquid media may not provide reliable knowledge about their abilities to utilize different sources in natural habitats. Last but not least, *in vitro* symbiotic propagation of orchids is typically carried out on solid media.

In this study, we investigated the capability, expressed as dry fungal biomass and radial growth rate, of nine fungal strains from the families Ceratobasidiaceae, Tulasnellaceae and Serendipitaceae isolated from European orchids to utilize different forms of C, N and P in both liquid and solid media. As a novelty, the study includes Tulasnellaceae members from a clade of important orchid symbionts that have not been so far investigated for their nutrient utilization. We hypothesised (H1) that the selected OMF isolates differ in their abilities to utilize nutrients and (H2) the isolate performance is affected by the media type.

## 2. Materials and Methods

### 2.1. Isolate Preparation

**Figure 1:**
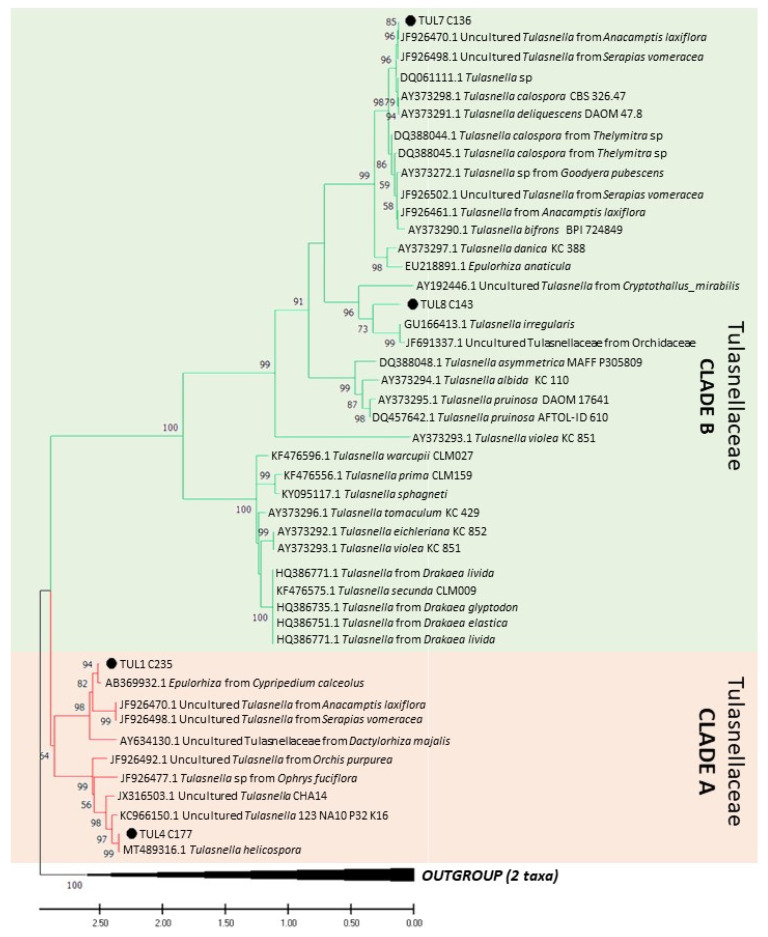

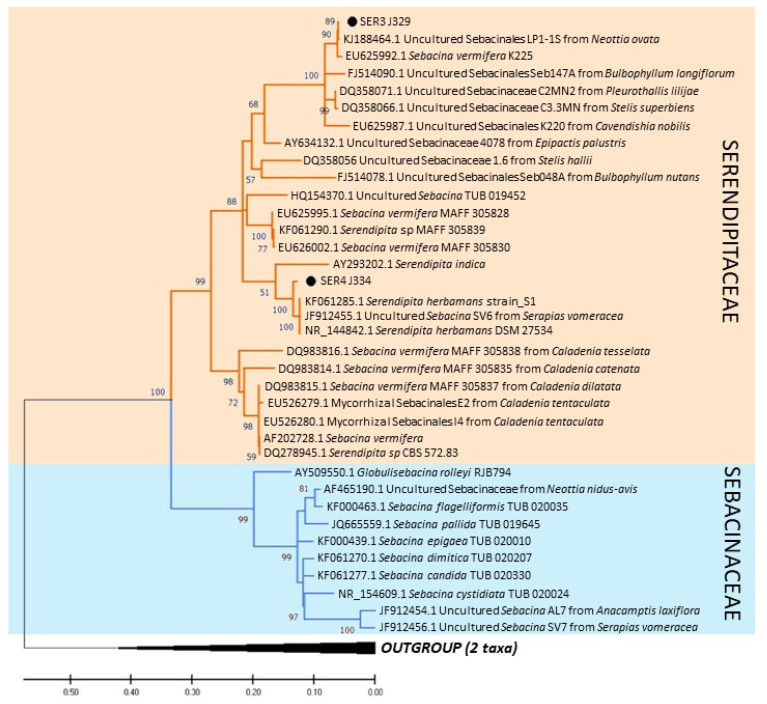

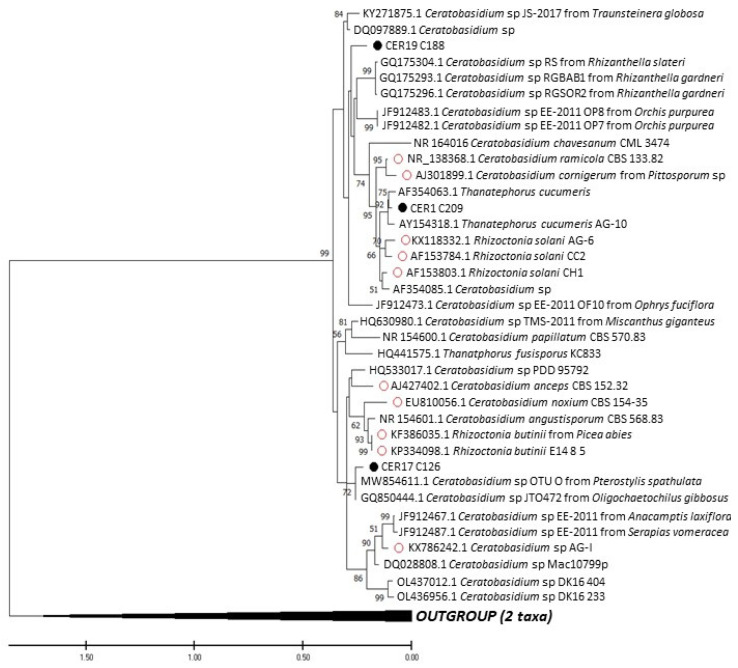
Phylogenetic trees of fungal isolates from families Tulasnellaceae (two lineages, clades A and B (**A**), Serendipitaceae (**B**), and Ceratobasidiaceae (**C**). The black circles represent the studied fungal isolates. The open red circles in the Ceratobasidiaceae tree represent fungal strains with reported pathogenic abilities.

Fungal isolates were obtained from mycorrhizal root sections of six orchid species collected in two species-rich grasslands in Southern France and the Czech Republic in May 2018 (Table 1). Roots of 10 individuals per species were washed under tap water, sterilised for 30 s with sodium hypochlorite (4.7%; further diluted to 1:5 with distilled water) and rinsed three times with distilled water. Hyphal coils, called pelotons, were teased out from the cortical cells of the sectioned tissue under a dissecting microscope and rinsed via serial dilution in four drops of distilled water in a laminar flow cabinet. Individual pelotons were micropipetted onto modified Melin–Norkrans (MMN) medium [33] (L^−1^): 1 g glucose, 0.3 g malt extract, 1 g KH_2_PO_4_, 0.25 g (NH_4_)_2_HPO_4_, 20.47 mg MgSO_4_ × 7 H_2_O, 5.7 mg ZnSO_4_ × 7 H_2_O, 1.3 mg CuSO_4_ × 5 H_2_O, 50 mg CaCl_2_, 25 mg NaCl, 20 mg FeCl_3_ × 6 H_2_O, 16 g agar and 50 mg Novobiocin added only at the isolation step. When fungal hyphae appeared from the pelotons, hyphal tips were subcultured to new plates and stored in the dark at 4 °C with routine subculturing every six months. The internal transcribed spacer (ITS) of nrDNA was amplified using ITS1/ITS4 [34] or ITS1OF/ITS4OF [35] primer pairs according to the protocol described in [36]. The amplicons were sequenced by Sanger sequencing by the commercial company SeqMe (Dobříš, Czech Republic). The sequences were grouped into operational taxonomic units (OTUs) based on commonly used 97% similarity over the ITS region using the clustering algorithm in TOPALi 2.5 [37]. Finally, nine isolates from different OTUs sorted into four fungal groups: Ceratobasidiaceae (CER), Serendipitaceae (SER), Tulasnellaceae clade A (TUL-A) and Tulasnellaceae clade B (TUL-B) were selected for the experiments (Table 1, see Figure 1 for phylogenetic relationships among the OTUs). Clade A comprised isolates related to *T. helicospora* (TUL4), representing an early diverging lineage in some other phylogenies of Tulasnellaceae [27,36], which has not yet been investigated for nutrient utilization ability. Clade B contained isolates related to *Tulasnella calospora* (TUL7) and *T. irregularis* (TUL8) species (sometimes called core Tulasnellaceae). Each fungal OTU was deposited in GenBank at NCBI (see Table 1). We also performed symbiotic seed germination to confirm that all used fungal isolates were mycorrhizal and able to trigger the germination of orchid species from which they had been obtained (our unpublished data).

### 2.2. Phylogenetic Analyses

Sequences obtained from fungal isolates isolated here from mycorrhizal root sections, as well as sequences publicly available in the National Center for Biotechnology Information (NCBI) GenBank database (www.ncbi.nlm.nih.gov/genbank; accessed on 1 November 2022) with high similarity (≥97.0%) regarding our strains and vouchered specimens were analysed to examine the phylogenetic similarity among them. The sequences of *Saitozyma pseudoflava* (MK050284.1) and *Trichosporon* sp. (DQ288848.2) were designated as outgroup taxa. Phylogenetic analyses were performed separately for each rhizoctonian family. Multiple sequence alignments were created with MAFFT v7.310 using the L-INS-i strategy [38]. Alignments were visualized and manually trimmed using BioEdit Sequence Alignment Editor [39] to ensure the best common coverage after alignment. Phylogenetic and molecular evolutionary analyses were conducted on MEGA v. 11 [40] using a maximum-likelihood (ML) method coupled with a Kimura two-parameters model and gamma distribution with invariant sites (G + I). To assess the relative robustness of branches, the bootstrap method was used with 1000 replicates [41] and values ≥50% are shown on the phylogenetic tree.

### 2.3. Experimental Media

At the beginning of the experiment, the isolates were transferred to water agar (WA; 16 g/L; Agar-Agar, Type I, HiMedia) to deplete possible food reserves accumulated in the hyphae. Subsequently, plugs excised with a sterilised cork borer (ø 5 mm) from the edge of the growing fungal colony served as an inoculum of solid or liquid medium with different C, N and P substrates added. The basal medium contained (L^−1^) 5 g glucose, 0.3 g KH_2_PO_4_, 0.25 g (NH_4_)_2_HPO_4_, 0.14 g MgSO_4_ × 7 H_2_O, 5.7 mg ZnSO_4_ × 7H_2_O, 1.3 mg CuSO_4_ × 5H_2_O, 50 mg CaCl_2_, 25 mg NaCl, 12.5 mg ferric EDTA, 0.13 mg thiamine HCl, 0.2 mg PABA, and 16g agar in case of solid medium [22]. Prior to autoclaving, the pH of the medium was adjusted to a range 4.7–5.5. Compounds containing carbon (glucose), nitrogen ((NH_4_)_2_HPO_4_) and phosphorus (KH_2_PO_4_ & (NH_4_)_2_HPO_4_) were replaced by the tested C, N and P sources, respectively, for each treatment. All the following treatments included four replicates and a control treatment free of tested source compound. For detailed medium composition of each treatment see Table S1.

#### 2.3.1. Carbon Sources

The ability of OMF to utilize various C substrates was tested using the medium containing one of the following 10 C sources: monosaccharides (glucose, galactose), disaccharides (cellobiose, trehalose), polysaccharides (pectin, xylan, starch, carboxymethylcellulose (CMC), and cellulose) and phenolic C (lignin) (for details, see Table S1). Mono- and disaccharides were sterilised through a Millipore filter (pore ø 22 µm, Merck Millipore Ltd., Darmstadt, Germany) prior to addition into the sterile basal medium. Polysaccharide CMC for solid medium was dissolved in 300 mL of distilled water (dH_2_O) and stirred with a magnetic stirrer at 50 °C, sterilised separately (autoclave cycle for 15 min at 121 °C) and subsequently added into the sterile basal medium under a laminar flow bench. For the liquid medium, CMC was first dissolved in dH_2_O at 50 °C using a stirrer and then added into the basal medium prior to autoclaving in a standard autoclave cycle (for 45 min at 121 °C). Water-insoluble cellulose and lignin were mixed and sterilised with basal medium in a standard way. For all C sources, the final C concentration was 2 g/L. In the case of solid medium, the amount of C contained in the agar itself was neglected in further analyses.

#### 2.3.2. Nitrogen Sources

The ability of OMF to utilize various N sources was tested using the medium containing one of following nine N sources: inorganic (NaNO_3_, (NH_4_)_2_HPO_4_, (NH_4_)_2_SO_4_), organic (CH_4_N_2_O, amino acids (L-glutamine, L-arginine, glutamic acid, glycine)), and casein enzymatic hydrolysate (N-Z-amine) (Table S1). All N sources are soluble in water and were therefore filter-sterilised and added into the medium after autoclaving. Only the treatment with glutamic acid required gentle heating of the solution prior to filtration to increase solubility. The exception was the treatment containing (NH_4_)_2_HPO_4_, where this N source was omitted from the basal medium composition and substituted by NaH_2_PO_4_ × 2H_2_O as P source (0.295 g/L). For all N sources, the final N concentration was 53 mg/L.

#### 2.3.3. Phosphorus Sources

The ability of OMF to utilize various P sources was tested using the medium containing one of following three P sources: inorganic (NaH_2_PO_4_ × 2H_2_O), organic (phytic acid, DNA from herring sperm) (Table S1). Phytic acid was dissolved in 10 mL of basal medium with the pH fixed in range from 4–4.5 prior its addition into the rest of the sterile basal medium through a Millipore filter. The DNA was first soaked in 70% ethanol for 48 h on a Petri plate at 4 °C. Subsequently, the alcohol was allowed to evaporate under the flow bench, and the sterile DNA was transferred into the sterile basal medium heated to 80 °C, where it dissolved. Phosphorus-containing compounds KH_2_PO_4_ and (NH_4_)_2_HPO_4_ were omitted from the basal medium and substituted by (NH_4_)_2_SO_4_ and KCl as N and potassium (K) sources, respectively. For all P sources, the final P concentration was 60 mg/L.

#### 2.3.4. Solid Cultures

The plastic Petri plates (ø 9 cm) containing ~ 25 mL of the experimental medium were overlaid with sterilised cellophane membrane (ø 7.5 cm; membrane ‘Celofán’ made from highly pure chemically regenerated cellulose, V+L MAIS Ltd., Zlín, Czech Republic). Each plate was inoculated with a 5-mm-diameter WA plug with mycelium and incubated at 20 °C in the dark until the mycelium covered the membrane and reached a similar diameter. Prior to mycelium harvesting, each plate was photographed to determine the radial growth rate (mm/day) of each fungus from the colony radius using ImageJ v. 1.52 [42]. Subsequently, the WA plug was removed, and the membrane with mycelium was transferred into a 50-mL Falcon tube filled with tap water. Tubes were submerged in a Water Bath Sonicator (SONOREX RK52, KRAINTEK 2^®^, Bandelin electronic GmbH & Co. KG, Berlin, Germany) for approx. 5 min. The detached mycelium was transferred into a pre-weighted aluminium dish (ø 25 mm), oven-dried for 3 h at 80 °C and weighted on a microbalance (MT5, METTLER TOLEDO, Columbus, OH, USA, d = 1 µg). The fungal biomass weight was subtracted from the dish weight and divided by the number of days in cultivation to obtain fungal biomass production rate (mg/day).

#### 2.3.5. Liquid Cultures

##### Determination of Exponential Growth Phase of the Isolates

The capacity of OMF to utilize C, N and P sources was assessed in the period of the exponential growth phase, determined by growing each isolate in MMN liquid medium in three replicates. Thirty mL of medium was poured into a 50-mL Cell culture tube with a filter screw cap (CELLSTAR^®^, CELLreactor™, Greiner Bio-One GmbH, Kremsmünster, Austria). Subsequently, each tube was inoculated with two 5-mm-diameter WA plugs with mycelium and incubated statically at 20 °C in the dark. Fast-growing fungi (CER1, CER19, TUL7, TUL8) and slower-growing fungi (CER17, TUL1, TUL4, SER3, SER4) were harvested every 2 and 4 days, respectively, with 10 harvesting events for either group. Harvesting involved filtering liquid cultures through a 100-µm nylon mesh with the washed hyphae transferred onto a pre-weighed aluminium dish, then dried for 3 h at 80 °C. The biomass included the WA plugs used for inoculation, as they could not be efficiently separated from the mycelium. The dry biomass weight was determined on the microbalance. The fungal growth curve was obtained by plotting the biomass of the OMF against time, and the exponential phase of the OMF was determined based on the growth curve. As the mycelial growth rates for OMF used in this trial varied significantly, the length of the incubation period was adjusted to ensure that all treatments were carried out during each isolate’s exponential growth phase at the time of harvest, which was standardised to approximately 10 mg dry fungal biomass. From the shortest to the longest incubation period, TUL7, CER19, TUL8, CER1, SER4, CER17, TUL1, SER3 and TUL4 were incubated respectively for 19, 21, 23, 25, 27, 29, 33, 33 and 39 days.

##### Liquid Culture Experiment

The experimental medium enriched with individual C, N or P sources was poured into tubes, inoculated with a fungus and incubated statically at 20 °C in the dark for the period determined for each isolate. Subsequently, the cultures were processed as described above.

### 2.4. Statistical Analysis

All data were log-transformed and analysed with the R statistical software version 3.5.2 [43]. The dry fungal biomass (mg/day) and the radial growth rate (mm/day, solid medium only) of each isolate on individual C, N and P substrates were evaluated using analysis of variance (ANOVA) with subsequent post-hoc tests by comparing the experimental plates against the control plates. The pattern in nutrient utilization among the four fungal groups (CER, SER, TUL-A and TUL-B) was visualised by principal components analysis (PCA) using log-transformed data and the function *rda* in the *vegan* package [44]. The effects of the source type (carbon, nitrogen and phosphorus) and the fungal group on fungal biomass were evaluated using the generalized linear mixed-effect model (GLMM), in the *lme4* package [45], applying the likelihood-ratio test (LRT) with assumed gamma distribution of random variation. The source type and the fungal group were defined as fixed factors, whereas the isolate identity and the C, N or P substrates represented random factors. We performed multiple comparisons of (i) the fungal groups within each source type and (ii) the source types within each fungal group.

## 3. Results

### 3.1. Fungal Biomass Production in Solid and Liquid Media 

**Figure 2:**
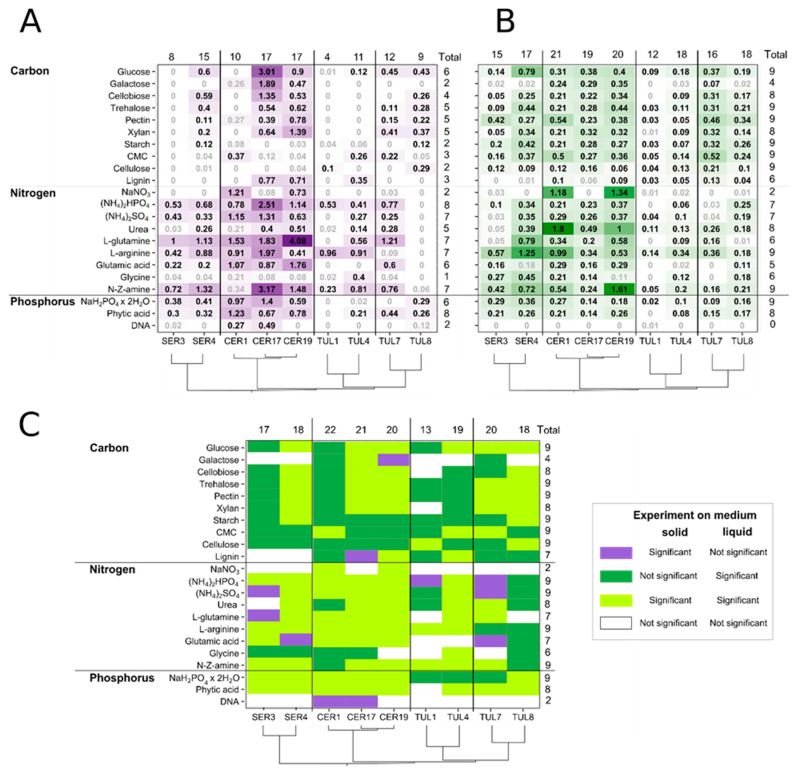
Fungal biomass production (in mg/day; mean value) of nine fungal isolates grown on C, N and P substrates in solid (**A**) or liquid media (**B**). The biomass production on a particular substrate was subtracted from the substrate-free control (values significant at *p* < 0.05 are denoted in bold). The colour shades indicate the amount of biomass increase. (**C**) Comparison of significant biomass increases between solid and liquid media. The phylogenetic tree based on ITS sequences shows genetic relatedness of the isolates (see Figure 1). The values on the margins of the chart represent the total numbers of treatments (on the top) or isolates (on the right) in which a significant biomass increase was induced at least in one medium. See Table S1 for details of each substrate.

**Figure 3:**
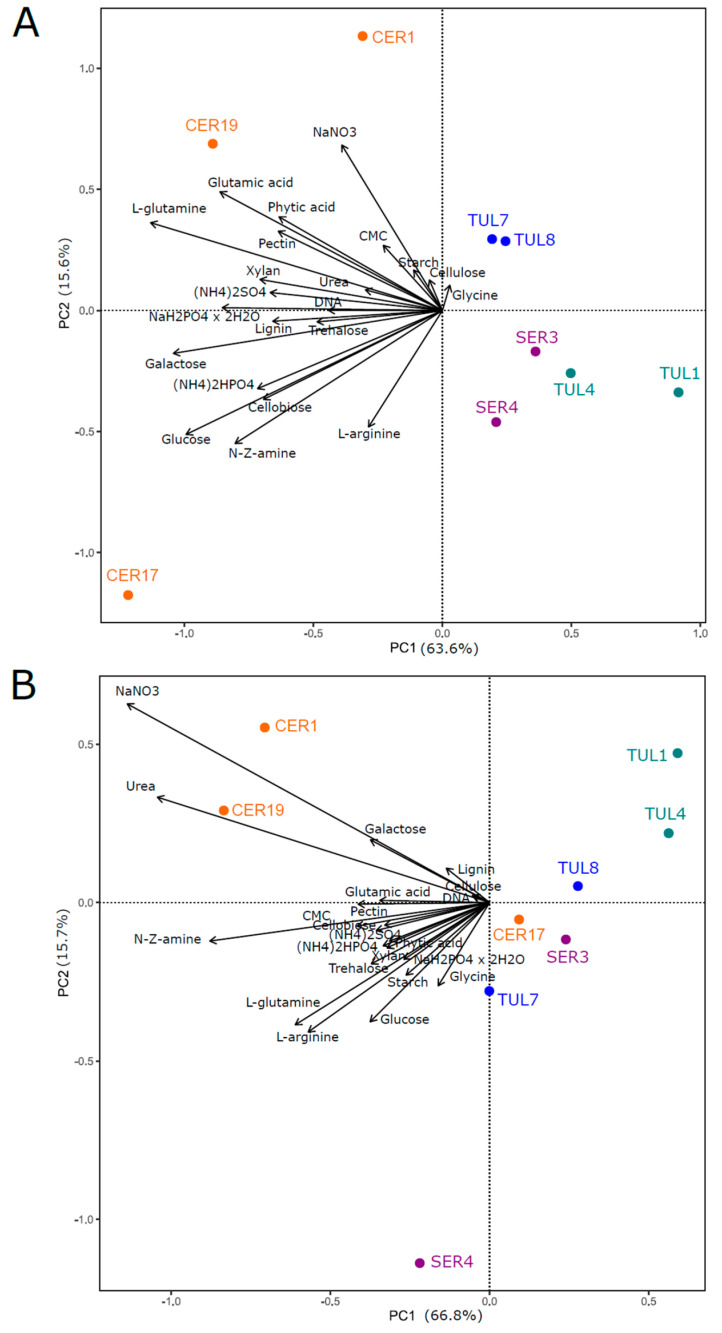
Principal components analysis of the fungal biomass of nine fungal isolates cultivated in solid (**A**) or in liquid (**B**) media, containing the 22 different carbon, nitrogen, and phosphorus sources (see Table S1 for details). The colour of the isolates indicates the fungal group: orange, purple, blue and green for Ceratobasidiaceae (CER1, 17, 19), Serendipitaceae (SER3, 4), Tulasnellaceae clade A (TUL1, 4) and Tulasnellaceae clade B (TUL7, 8), respectively.

In general, fungal biomass production was higher in solid medium than in liquid medium for all isolates on most tested substrates (Figure 2A,B). Fungal group had a significant effect on fungal biomass production in both medium types, but this effect differed according to the interaction of the fungal group with a particular source type (C, N, P) (Table S2). While Ceratobasidiaceae produced a significantly higher fungal biomass than Tulasnellaceae clade A on all source types and both media types, the larger biomass of Ceratobasidiaceae than that of Serendipitaceae was achieved only in liquid medium (Table 2). Tulasnellaceae clade B produced a significantly higher fungal biomass than Tulasnellaceae clade A on all source types and both medium types, except for C source in liquid medium (Table 2). On solid medium, the variability in fungal biomass production between fungal groups and isolates was driven mainly by N substrates, and some C and P substrates such as glucose, galactose, inorganic phosphate and phytic acid (Figure 3A). In liquid medium, the major variability drivers were N substrates, particularly urea and sodium nitrate (Figure 3B).

This pattern, however, changed when we compared the isolates’ biomass grown on a particular substrate with that of the substrate-free control (Figure 2). While the fungal biomass produced on substrates in the liquid medium was mostly significantly higher than the biomass in the control, on the solid medium, the biomass frequently did not significantly differ between the substrate and the control. Moreover, some isolates showed no or limited biomass production on solid medium with particular nutrient sources. 

### 3.2. Carbon Sources

*Solid medium:* Fungal isolates CER1, SER3, TUL1 and partially TUL4 (both Tulasnellaceae clade A) showed no or low capability to metabolise C substrates compared with the C-free control treatment (Figure 2A and Figure S1, Table S3). Isolates CER17 and CER19 effectively used all C substrates except for polysaccharides starch, CMC and cellulose. On the other hand, CER1 grew significantly only on CMC-rich substrate. While SER3 utilized none of the tested C substrates, SER4 produced significant biomass amounts on glucose-, cellobiose-, trehalose-, pectin-, xylan- and starch-rich medium. All Tulasnellaceae, except for TUL1, effectively metabolised glucose. Nonetheless, only TUL1 and TUL8 were able to degrade cellulose. The use of other C-sources by Tulasnellaceae was scarce, although TUL7 and TUL8 were able to produce significant biomass amounts on glucose, trehalose, pectin and xylan. Lignin use was demonstrated in the case of CER17, CER19 and SER4. 

*Liquid medium:* The mean biomass increase of Ceratobasidiaceae was significantly higher compared to that of other fungal groups (Figure 2B and Figure S2, Table S2). Except for the non-significant biomass increase of CER17 on lignin-rich medium, all Ceratobasidiaceae effectively used all tested C sources (Table S3). Both Serendipitaceae OTUs showed difficulties in metabolising galactose and lignin. In general, Tulasnellaceae isolates were able to effectively utilize all tested C-substrates except for galactose, cellobiose and xylan. 

### 3.3. Nitrogen Sources

In general, Ceratobasidiaceae demonstrated the highest biomass production detected in both medium types (Figure 2A,B). Some isolates, however, showed preference for either liquid or solid medium with a particular N-source. For example, CER1 produced the greatest fungal biomass when it metabolised urea (1.8 mg/day) in liquid medium, whereas its biomass increase on solid medium was not significantly different from that of the substrate-free control (Figures S3 and S4). Isolate CER19 gained 4.08 mg/day of biomass in L-glutamine-rich agar, which was the highest overall biomass production recorded, whereas in liquid medium, it reached only 0.58 mg/day (Figures S3 and S4). Tulasnellaceae clade A showed the lowest biomass increase of all fungal groups (Figure 2A,B, Table 2). A common trend of Serendipitaceae, Tulasnellaceae and CER17 in both media was the disability to digest nitrate (NaNO_3_).

*Solid medium:* The fungal biomass obtained on all tested N substrates differed significantly from that obtained in the substrate-free control, except for TUL8 with poor biomass production on all N substrates (Figure 2A and Figure S3, Table S3). However, there were apparent differences in substrate utilization among fungal groups and isolates. Ammonium phosphate ((NH_4_)_2_HPO_4_), L-glutamine, L-arginine and N-Z amine were the most suitable substrates for the growth of all fungal groups, with some exceptions, mainly from the Tulasnellaceae group. In contrast, glycine resulted in no or minimal biomass production except in TUL4. Isolate TUL8 did not effectively utilize any of the tested N substrates. 

*Liquid medium:* All Ceratobasidiaceae effectively used all N sources except nitrate and glycine in case of CER17 and CER19, respectively (Figure 2B and Figure S4, Table S3). Isolate SER4 did not grow well on glutamic acid substrate and SER3 on ammonium sulphate (NH_4_)_2_SO_4_, urea and L-glutamine, respectively. Tulasnellaceae clade A and TUL7 produced no or small amounts of biomass on glutamic acid and (NH_4_)_2_SO_4_, respectively, and TUL1, together with TUL7, lacked the ability to metabolise amino acid glycine.

### 3.4. Phosphorus Sources

Two common trends were observed in both solid and liquid media: (i) inability to utilize DNA, except for CER1 and CER17 on solid medium; (ii) effective utilization of inorganic phosphate NaH_2_PO_4_ × 2H_2_O and organic phytic acid substrates by all isolates, except for Tulasnellaceae clade A and TUL7 from clade B on the NaH_2_PO_4_ × 2H_2_O substrate and TUL1 from clade A on phytic acid on solid medium (Figure 2A,B, Figures S5 and S6, Table S3).

### 3.5. Comparison of Radial Growth Rates on Solid Medium

When comparing the radial growth rates of the isolates on a particular source type (C, N, P) against substrate-free controls, we only observed significant growth of Ceratobasidiaceae and TUL8 isolates on N and P sources, respectively (Table S4). Specifically, most Ceratobasidiaceae isolates showed significant radial growth rates on NaNO_3_, (NH_4_)_2_HPO_4_, urea, L-glutamine, L-arginine; Serendipitaceae and Tulasnellaceae isolates showed occasional significant growth on a particular substrate, but without a consistent pattern (Table S4).

## 4. Discussion

Our data provided substantial evidence about the metabolic behaviour of nine isolates from the families Ceratobasidiaceae, Tulasnellaceae and Serendipitaceae cultivated simultaneously on solid and in liquid media. This is a unique feature because most studies have provided data from investigation of one orchid fungal family only [21,24,46] or several families but grown in liquid cultures only and a limited number of isolates (Nurfadilah et al. (2013) [22]—four OTUs in total). We also demonstrated a strong effect of Tulasnellaceae phylogenetic affinity (clade A vs. clade B) on nutrient acquisition, with isolates from clade A growing much slower, producing lower amount of biomass and utilizing a lower spectrum of nutrients than isolates from clade B. Isolates from clade A were studied for nutrient utilisation for the first time, which extends our knowledge on the fungal traits of the family Tulasnellaceae. In addition, our fungal isolates were obtained from six terrestrial orchid species from the northern hemisphere, thus our study broadens the current view of OMF nutrient utilization compared to available studies from Australia [22,23,24] and provides knowledge applicable to ex situ conservation of European orchids.

### 4.1. Carbon Utilization

After subtracting the biomass amount from that of the C-free control, almost all tested fungal OTUs showed significant biomass production in liquid medium, whereas the production on solid medium was only rarely significantly different from that of the control. Such a discrepancy could be explained (i) by the utilisation of a cellophane membrane as a C source by mycelium, and/or (ii) by the presence of additional C sources in the agar of the solid medium, such as cellulose, agarose and agaropectin. Indeed, most isolates were able to produce some biomass on solid medium and we have sometimes noticed visual decomposition of the cellophane membrane. One possibility to avoid the use of a cellophane membrane could be analysis of the ergosterol content [23,46,47], which is a fungus-specific membrane lipid. The effect of agar itself can be solved only by using liquid medium.

On solid medium, fungal biomass production on most mono-, di- and polysaccharides was significantly higher than that of the C-free control, mainly in Ceratobasidiaceae and Tulasnellaceae clade B and SER4 isolates. The isolate CER17 formed the greatest fungal biomass of all isolates on the monosaccharide’s glucose (3.01 mg/day) and galactose (1.89 mg/day), which is congruent with the findings of Nurfadilah et al. (2013) [22], demonstrating a high biomass production of a *Ceratobasidium* sp. (DQ028808.1; Figure 1C) on these carbon sources in liquid medium. In liquid medium, glucose, cellobiose, trehalose, pectin, xylan, starch, CMC and cellulose were the C sources utilised by the majority of the tested isolates. Generally, the polysaccharides pectin and CMC promoted the highest biomass production [48], while other studies [21,22,23] identified the polysaccharide xylan as a more efficient C-source. 

The fungal groups largely varied in C-source utilization. Ceratobasidiaceae could metabolise the widest range of examined sugars, which might be related to their different nutritional modes, known as pathogenic, saprotrophic, ectomycorrhizal or endophytic fungi [7,29]. Veldre et al. (2013) [7] have suggested that autotrophic orchids form mycorrhizal associations with any available soil Ceratobasidiaceae, regardless of any pathogenicity they may have that might be restricted by the plant host, e.g., by the production of antifungal compounds [49]. Indeed, strains with confirmed pathogenicity are scattered along the Ceratobasidiaceae phylogenetic tree (Figure 1C) and in a recent study by Freestone et al. (2021) [50] one OTU appeared that contained both pathogenic and OMF forms. Tulasnellaceae clades differed significantly in their utilisation of tested C sources, with Tulasnellaceae clade B showing a significantly higher biomass production on the majority of the tested carbohydrates compared with clade A. Genomic and enzyme expression analyses of *T. calospora* from Tulasnellaceae clade B (96.3% identity between *T. calospora* voucher AY373298.1 and our TUL7) revealed numerous genes encoding the activation of carbohydrate-active enzymes (CAZymes), responsible for the degradation of lignocellulolytic compounds in plant cell walls [2,11]. However, the question of why Tulasnellaceae clade A does not use carbohydrates as efficiently as the sister group still needs to be explored. Serendipitaceae showed no or restricted biomass production on lignin and galactose, as shown in other studies [22,23,24]. Among micro-organisms, fungi are the most efficient lignin decomposers, but OMF from the family Ceratobasidiaceae have shown limited ability to utilize complex aromatic compounds, such as tannins, compared to ericoid mycorrhizal fungi ([21], and references therein); moreover, the extent of tannin degradation strongly varied among the isolates.

### 4.2. Nitrogen Utilization

The studied OMF isolates were able to utilize all tested N sources. Nitrogen is an essential element for fungal growth and biomass production [51]. It can be obtained either from mineral or organic compounds but is ultimately converted into inorganic NH_4_^+^ and amino acids (glutamate, glutamine, arginine), serving as storage N-containing compounds of the cells used in biosynthetic reactions [52]. Mycorrhizal fungi help their host plants with nitrogenous compound acquisition, and in the case of orchids, N represents the probable major nutrient transferred from OMF to the plants, as orchid tissue is highly N-enriched [53]. Cameron et al., (2006) [54] experimentally demonstrated that 78% of extra-radically supplied ^15^N-labelled glycine accumulated in the mycelial biomass of *Ceratobasidium cornigerum* compared to 20% in the roots and <3% in shoot tissues of the terrestrial orchid *Goodyera repens*.

Here, inorganic ammonium-containing substrates ((NH_4_)_2_HPO_4_, (NH_4_)_2_SO_4_) were effectively assimilated by the majority of the tested OTUs, confirming that directly accessible ammonium is a preferable N source for most filamentous fungi [52]. The uptake is mediated via specific ammonium membrane transporters, recognized in OMF, specifically in *Serendipita vermifera* and *S. bescii* [25,55]. Ammonium utilization by OMF isolates has been demonstrated in several *in vitro* studies [22,48]; however, its effective assimilation significantly varied among Serendipitaceae OTUs [24] or in *T. calospora* strains [25,56].

On the other hand, inorganic nitrate (NO_3_^−^) and complex organic compounds are less frequently assimilated by fungal hyphae [57]. In our study, nitrate (NaNO_3_) stimulated biomass production only in two Ceratobasidiaceae isolates, CER1 and CER19, and the remaining OMF showed very poor or no growth. Similar findings have been obtained in other studies [22,56,58], where effective nitrate use was observed for *Ceratobasidium* spp. but not for other OMF genera. Vogt-Schilb et al. (2020) [59] demonstrated a higher abundance of Ceratobasidiaceae OTUs in meadows restored from arable fields than in natural grasslands, which aligns with the ruderal characteristics of the family and its ability to utilize nitrates from inorganic fertilisers. Recently, Figura et al. (2021) [60] observed no inhibitory effect of increasing nitrate concentrations on the germination success of *Dactylorhiza majalis* symbiotically cultivated with *Ceratobasidium* sp., but the authors found a strong inhibition when germinated with *Serendipita* sp. and *Tulasnella* spp. The inability of *Serendipita indica* and *T. calospora* to assimilate nitrate was confirmed when no functional nitrate transporters were identified [25,61]. The ability to utilize nitrates does not seem to be inherited by the whole Ceratobasidiaceae family, as isolate CER17 did not show this ability. This variation in nitrate utilization needs to be explored for a broader number of Ceratobasidiaceae isolates at both inter- and intra-specific levels.

Not all organic N-containing compounds tested in this study were good sources for utilization by OMF isolates. The simple amino acids L-arginine and L-glutamine, together with peptone N-Z amine, were effectively metabolised by most tested isolates, whereas glycine and glutamic acid were used poorly. Our findings agree with Stephen and Fung (1971) [62] and Nurfadilah et al., (2013) [22], who observed reasonable fungal biomass production on L-arginine and L-glutamine, but the greatest biomass yield was gained on glutamic acid-rich medium. Contrarily, Hadley and Ong (1978) [56] found glycine and urea to be suitable N sources for a *T. calospora* isolate, whereas L-glutamine and L-arginine were less suitable. Another isolate of *T. calospora* produced the highest biomass on L-glutamine, followed by glutamic acid [25]. Serendipitaceae isolates had the capacity to assimilate various N organic compounds such as amino acids, urea and L-arginine, but showed strong influence of isolate identity towards a particular organic N source [24,55]. In addition, we found substantial differences in N utilization by OMF when grown in liquid or on solid media. Most noticeably, urea and glycine were more efficiently utilized in liquid medium and with a wider range of tested fungal isolates. The differences between media types might stem from limited mobility of nutrients in solid media in comparison to liquid cultures [63].

### 4.3. Phosphorus Utilization

Most tested isolates effectively utilized inorganic orthophosphate (NaH_2_PO_4_ × 2 H_2_O) and phytic acid in both liquid and solid media, but, with some exceptions, did not utilise DNA (Figure 2A,B). Inorganic orthophosphate is a primary source of P and freely accessible to soil micro-organisms and plants, however, due to insufficient reserves in soil, it needs to be continually replenished by the mineralisation of organic P [64]. The uptake of orthophosphate from the soil environment by mycorrhizal fungi is mediated by specific membrane transporters, mainly located in the extra-radical mycelium [65]. They have been described in arbuscular and ectomycorrhizal fungi [66,67] and recently also in OMF, namely *T. calospora* and *S. vermifera* [2,68]. We observed effective NaH_2_PO_4_ × 2 H_2_O utilisation by the Ceratobasidiaceae and Serendipitaceae OTUs in both medium types compared to the P-free control, similar to other OMF studies [22,69]. On the contrary, Serendipitaceae isolates produced low biomass amounts on NaH_2_PO_4_ × 2 H_2_O in liquid cultures, which has been attributed to the limited P availability in Australian soils [24].

Phytic acid and DNA are organic P sources which are not directly available for soil micro-organisms or plants, and their utilization is conditioned by the microbial secretion of extracellular hydrolytic enzymes [70]. Numerous fungal phytases catalysing the hydrolysis of abundant phytic acids belong to the family of acid phosphatases, which are highly activated in saprotrophic fungi [71]. In the present study, we observed the highly effective use of phytic acid by all fungal isolates in both medium types, except for TUL1. Similar results have been achieved by Nurfadilah et al., (2013) [22]; however, the *S. vermifera* isolate produced the lowest biomass amount. By testing numerous *Serendipita* isolates, Oktalira (2020) [24] observed significant differences in mean fungal biomass among isolates within the same OTU.

Nucleic acids with phospholipids form a minor part of soil organic P; they need to be degraded by phosphodiesterases to become bioavailable. In saprotrophic fungi, the activity of these enzymes is secured by a lower number of genes, resulting in lower activities of phosphodiesterases compared to acid or alkaline phosphatases [71]. In our study, we revealed no or limited use of DNA by OMF in both medium types, apart from CER1 and CER17 on solid medium. The effective utilization of DNA by some Ceratobasidiaceae might be the result of their wide ecological amplitude, including pathogenic behaviour. Other studies have shown an ability of OTUs from all three orchid mycorrhizal families to use DNA, albeit with an effectiveness lower than for other P sources [22,24].

### 4.4. Disputable Use of Radial Growth Rate

The radial growth rate (or colony diameter) is a popular indirect measure of filamentous growth on solid media [72,73,74]. While the biomass production rate of the tested isolates on solid medium was significant in most cases, comparisons of radial growth rates on substrate and substrate-free controls yielded mostly non-significant results. The reason for such a discrepancy likely stems from the behaviour of filamentous fungi, exploring the habitat with rapidly growing, sparsely branched hyphae [75]. Therefore, we recommend avoiding radial growth rate as a measure for the assessment of substrate utilisation efficiency in filamentous fungi.

## 5. Conclusions

In the present study, we found significant inter- and intra-familiar variability and a strong effect of Tulasnellaceae phylogeny on C, N and P acquisition. The nutrient resource abilities of isolates strongly differed when grown in liquid or solid media, therefore we urge caution when generalizing knowledge from liquid cultures to ecology of OMF in situ. The main gaps in our current knowledge on OMF are in the ecology of enzyme production under various natural or semi-natural conditions, which could be explored in future by approaches used for arbuscular mycorrhizae [76,77].

## Figures and Tables

**Table 1:** Description of fungal isolates. Fungal groups: SER—family Serendipitaceae, CER—family Ceratobasidiaceae, TUL-A and TUL-B—two clades within Tulasnellaceae, see Figure 1A–C. Sites of orchid host origin: CZ—Czech Republic (49.1292N; 13.6591E), F—France (43.9669N; 3.4028E). OTU—operational taxonomic unit.

OTU Code	Fungal Group Code	Orchid Host	Site	Genbank Accession Number	Genetic Similarity with the Nearest Taxonomically Informative Blast
CER1	CER	*Neotinea ustulata*	F	MZ502998	96.9% AF354063.1 *Thanatephorus cucumeris*
CER17	CER	*Anacamptis morio*	F	MZ502999	89.0% AJ427402.1 *Ceratobasisium anceps*
CER19	CER	*Ophrys sphegodes* *subsp. passionis*	F	MZ503000	91.2% DQ097889.1 *Ceratobasidium* sp.
SER3	SER	*Neottia ovata*	CZ	MZ503001	97.1% EU625992.1 *Sebacina vermifera*
SER4	SER	*Neottia ovata*	CZ	MZ503002	95.8% NR144842.1 *Serendipita herbamans*
TUL1	TUL-A	*Anacamptis pyramidalis*	F	MZ503003	97.9% AB369932.1 *Epulorhiza* sp.
TUL4	TUL-A	*Orchis mascula*	F	MZ503004	97.9% MT489316.1 *Tulasnella helicospora*
TUL7	TUL-B	*Anacamptis morio*	F	MZ503005	96.3% AY373298.1 *Tulasnella calospora*
TUL8	TUL-B	*Anacamptis morio*	F	MZ503006	76.4% GU166413.1 *Tulasnella irregularis*

**Table 2:** Generalized linear mixed-effect models (GLMMs) comparing fungal biomass grown on three source types (carbon, nitrogen, phosphorus) in both solid and liquid media. Tukey post-hoc multiple comparison tests of the four fungal groups: Serendipitaceae (SER), Ceratobasidiaceae (CER), Tulasnellaceae (two clades TUL-A and TUL-B), within each source type; * *p* < 0.05, ** *p* < 0.01, *** *p* < 0.001.

		Source Type								
		Carbon			Nitrogen			Phosphorus		
Media Type	Multiple Comparison Fungal Group vs. Source Type	Estimate (±SE)	z-Value	*p*-Value	Estimate (±SE)	z-Value	*p*-Value	Estimate (±SE)	z-Value	*p*-Value
Solid	CER vs. SER	0.37 (0.17)	2.10	0.15	0.26 (0.17)	1.47	0.45	0.11 (0.18)	0.62	0.92
	CER vs. TUL−A	0.78 (0.17)	4.38	<0.001 ***	1.01 (0.17)	5.64	<0.001 ***	0.55 (0.18)	2.98	0.01 *
	CER vs. TUL−B	0.14 (0.17)	0.82	0.84	0.44 (0.17)	2.49	0.060	−0.09 (0.18)	−0.49	0.96
	SER vs. TUL−A	0.41 (0.19)	2.09	0.15	0.74 (0.19)	3.80	<0.001 ***	0.44 (0.20)	2.15	0.14
	SER vs. TUL−B	−0.22 (0.19)	−1.15	0.65	0.18 (0.19)	0.93	0.79	−0.21 (0.20)	−1.02	0.74
	TUL−A vs. TUL−B	−0.63 (0.19)	−3.24	0.006 **	−0.56 (0.19)	−2.87	0.02 *	−0.65 (0.20)	−3.17	0.008 **
Liquid	CER vs. SER	0.95 (0.28)	3.38	0.003 **	0.88 (0.28)	3.09	0.01 *	1.38 (0.31)	4.36	<0.001 ***
	CER vs. TUL−A	1.35 (0.28)	4.80	<0.001 ***	1.36 (0.28)	4.74	<0.001 ***	2.90 (0.32)	8.95	<0.001 ***
	CER vs. TUL−B	0.77 (0.28)	2.74	0.030 *	0.15 (0.28)	0.54	0.95	1.13 (0.31)	3.59	0.002 **
	SER vs. TUL−A	0.39 (0.30)	1.27	0.58	0.48 (0.31)	1.54	0.41	1.51 (0.35)	4.30	<0.001 ***
	SER vs. TUL−B	−0.18 (0.30)	−0.59	0.93	−0.72 (0.31)	−2.31	0.09	−0.25 (0.34)	−0.73	0.88
	TUL−A vs. TUL−B	−0.57 (0.30)	−1.87	0.24	−1.20 (0.31)	−3.83	<0.001 ***	−1.76 (0.34)	−5.07	<0.001 ***

## Data Availability

Dryad repository DOI https://doi.org/10.5061/dryad.kwh70rz7s.

## Supplementary Materials

The following supporting information can be downloaded at: https://www.mdpi.com/article/10.3390/jof9010088/s1, Figures S1,S3,S5: Mean biomass (in mg/day) ±SD of the tested isolates in solid medium containing carbon, nitrogen and phosphorus sources, respectively; Figures S2,S4,S6: Mean biomass (in mg/day) ± SD of the tested isolates in liquid medium containing carbon, nitrogen and phosphorus sources, respectively; Table S1: Characteristics of studied compounds added into solid and liquid MMN media: carbon (C), nitrogen (N), and phosphorus (P) sources; Table S2: Generalized linear mixed models (GLMMs) testing the effects of the Fungal group, the Source type, and the interaction term by likelihood-ratio test (LRT) comparing with Chi2 distribution, Post-Hoc multiple comparison tests of the interaction term: testing the biomass differences among Fungal groups within a Source type; and testing the biomass differences among Source types within a Fungal group; Table S3: ANOVA with multiple comparisons of means: contrasts testing the biomass among all substrates against the substrate-free control in both solid and liquid medium types, and Dunnett’s Post-hoc tests: contrasts comparing the fungal biomass of particular C, N, P substrates with substrate-free control in both solid and liquid medium types; Table S4: ANOVA with multiple comparisons of means: contrasts testing the fungal growth rate among all substrates against the substrate-free control in both solid and liquid medium types, and Dunnett’s Post-hoc tests: contrasts comparing the fungal growth rate on particular C, N, P substrates with substrate-free control in both solid and liquid medium types.
