# Supplementary material for: Variability in Nutrient Use by Orchid Mycorrhizal Fungi in Two Medium Types
Source: J Fungi (Basel). 2023 Jan 6;9(1):88. doi: 10.3390/jof9010088 (PMC9865426; doi:10.3390/jof9010088)

Figure S1. Mean biomass (in mg/day) ± SD (n=3 or 4) of the tested isolates (for which the biomass was significantly different from the control, in purple) in solid medium containing 10 different carbon sources. Different letters indicate a significant difference in the biomass increase ($P < 0.05$) between the different isolates and per each substrate. Grey bars indicate isolates with no significant biomass increase.

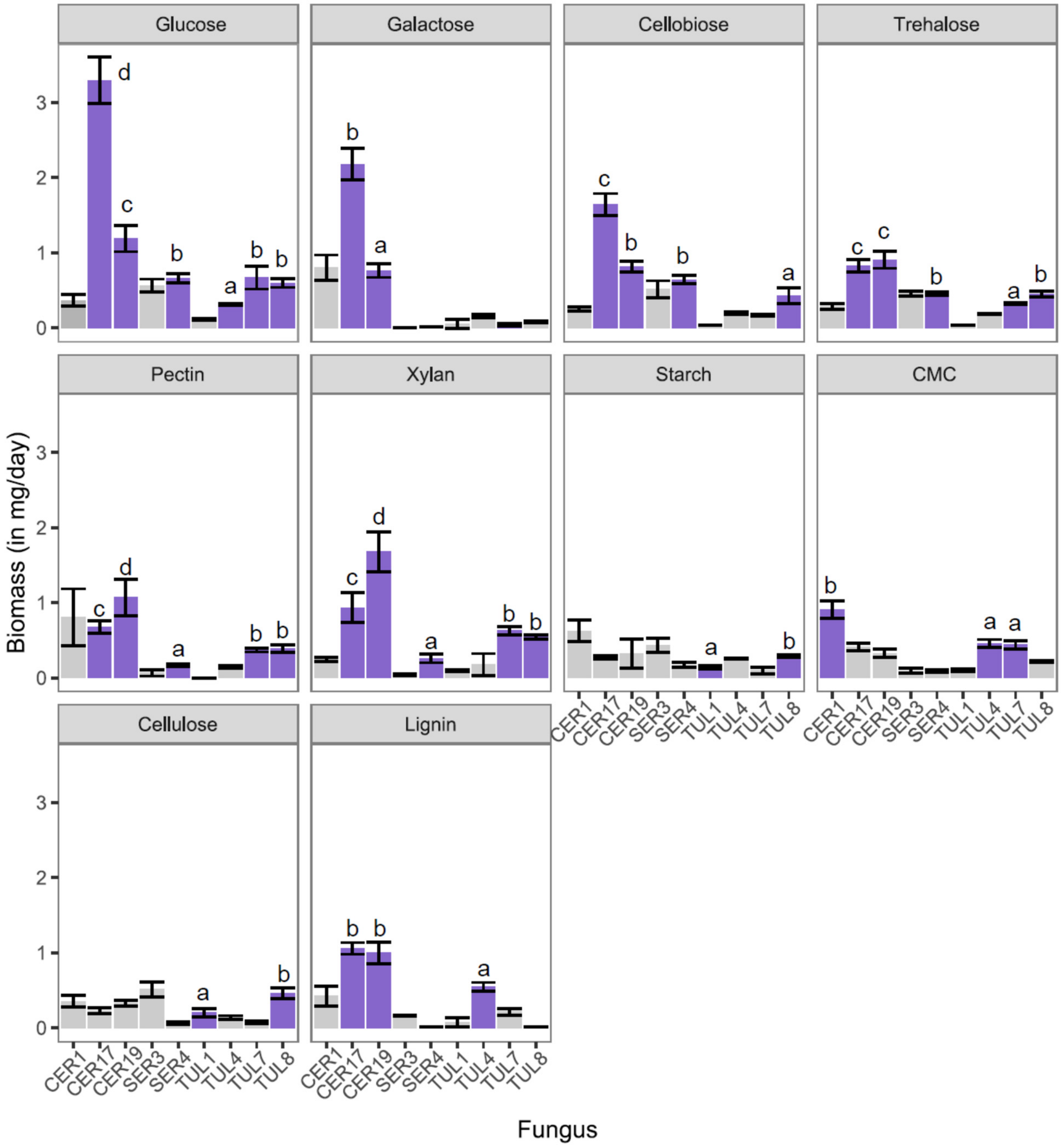

Figure S2. Mean biomass (in mg/day) ± SD (n=3 or 4) of the tested isolates (for which the biomass was significantly different from the control, in dark green) in liquid medium containing 10 different carbon sources. Different letters indicate a significant difference in the biomass increase ($P < 0.05$) between the different isolates and per each substrate. Grey bars indicate isolates with no significant biomass increase.

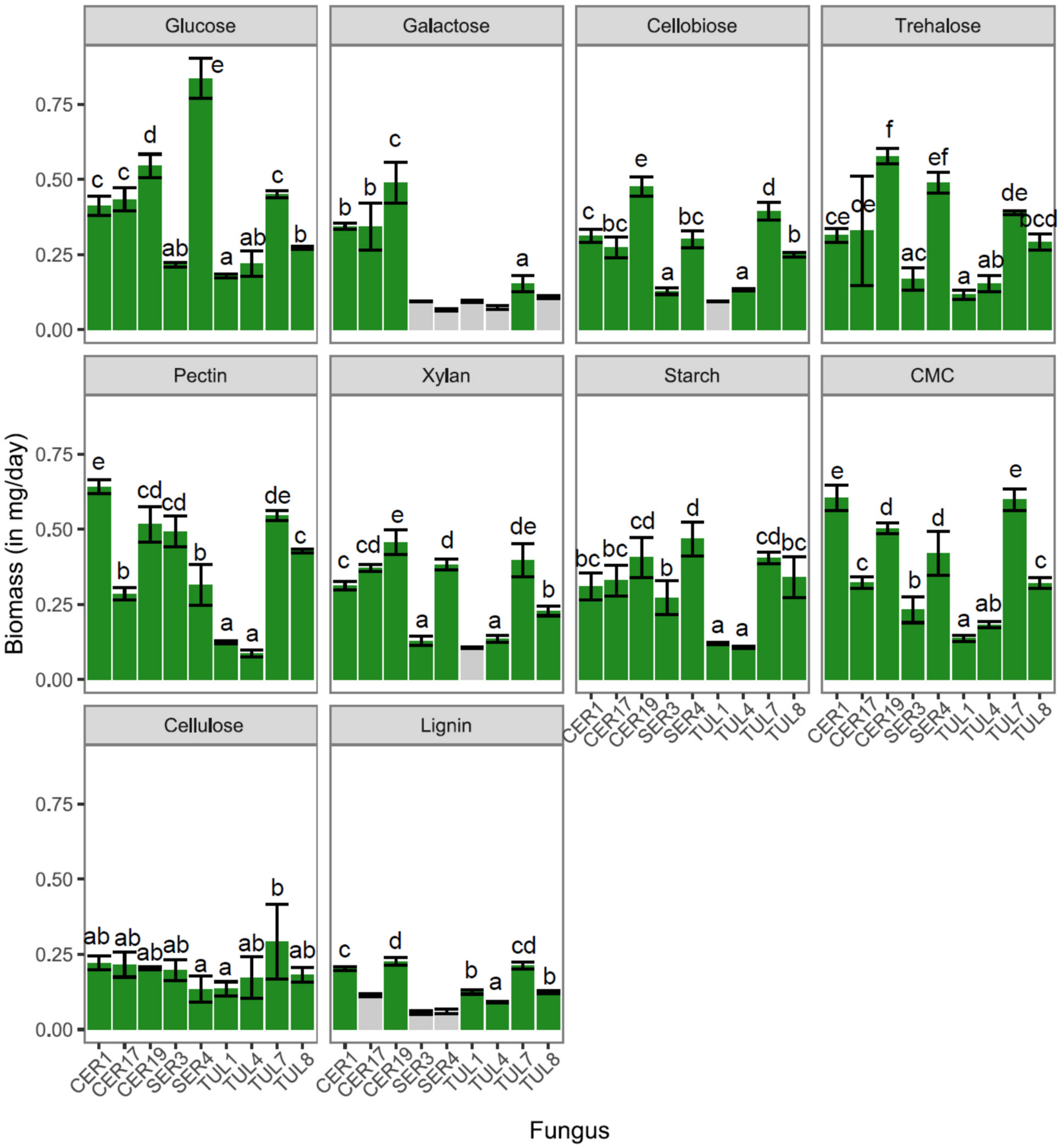

Figure S3. Mean biomass (in mg/day) ± SD (n=3 or 4) of the tested isolates (for which the biomass was significantly different from the control, in purple) in solid medium containing nine different nitrogen sources. Different letters indicate a significant difference in the biomass increase (P < 0.05) between the different isolates and per each substrate. Grey bars indicate isolates with no significant biomass increase.

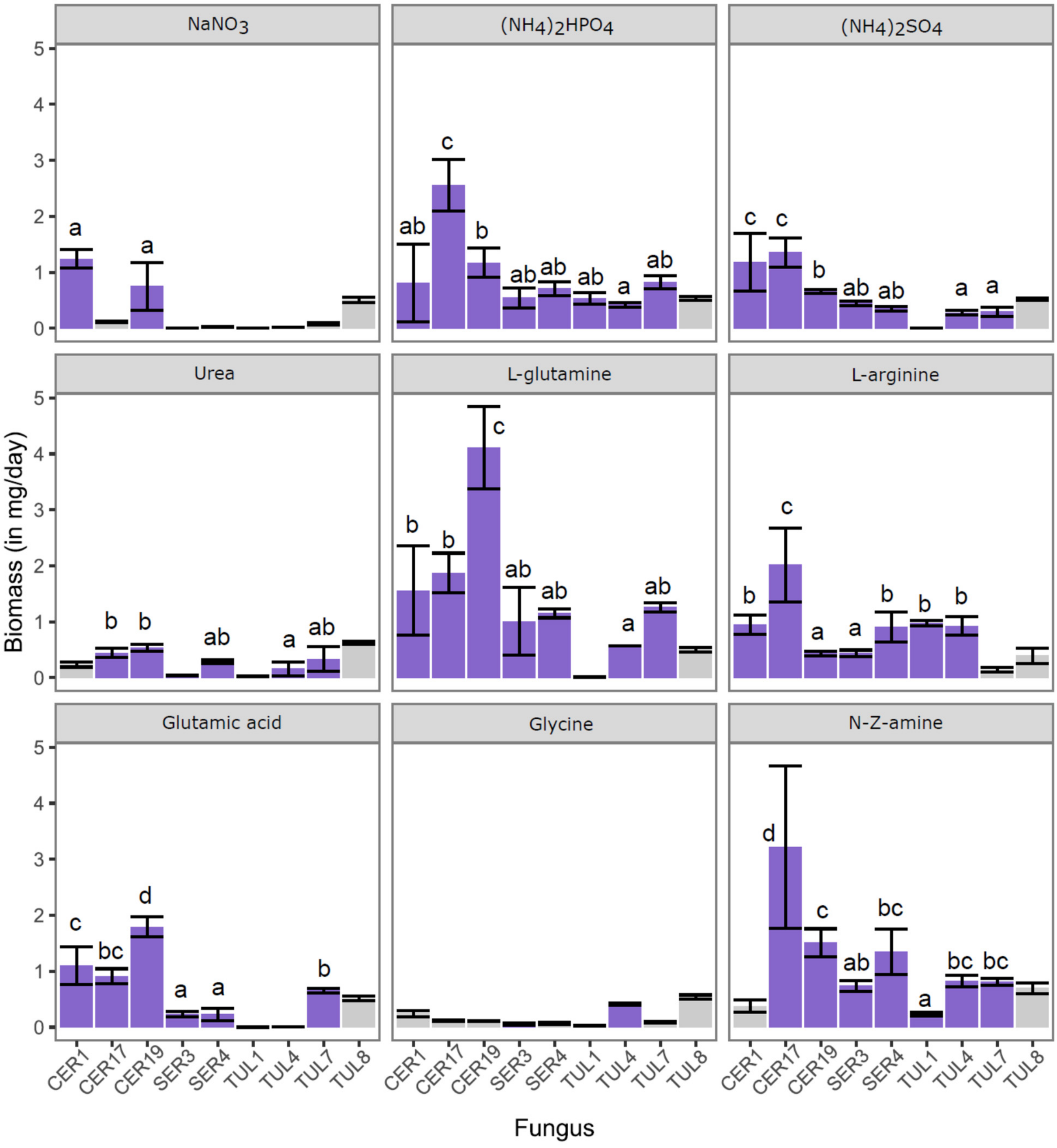

Figure S4. Mean biomass (in mg/day) ± SD (n=3 or 4) of the tested isolates (for which the biomass was significantly different from the control, in dark green) in liquid medium containing nine different nitrogen sources. Different letters indicate a significant difference in the biomass increase ($P < 0.05$) between the different isolates and per each substrate. Grey bars indicate isolates with no significant biomass increase.

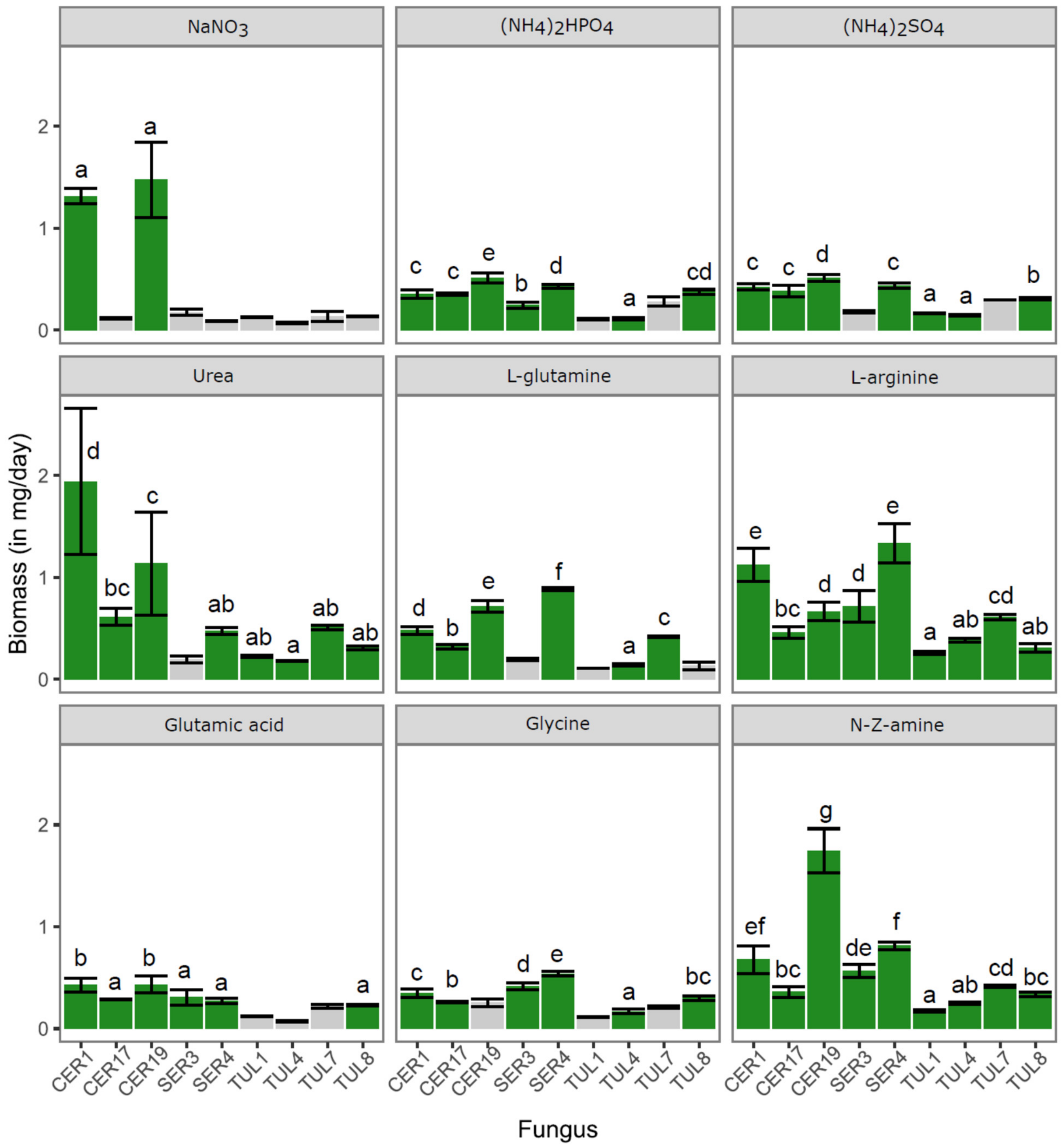

Figure S5. Mean biomass (in mg/day) ± SD (n=3 or 4) of the tested isolates (for which the biomass was significantly different from the control, in purple) in solid medium containing three different phosphorus sources. Different letters indicate a significant difference in the biomass increase ($P < 0.05$) between the different isolates and per each substrate. Grey bars indicate isolates with no significant biomass increase.

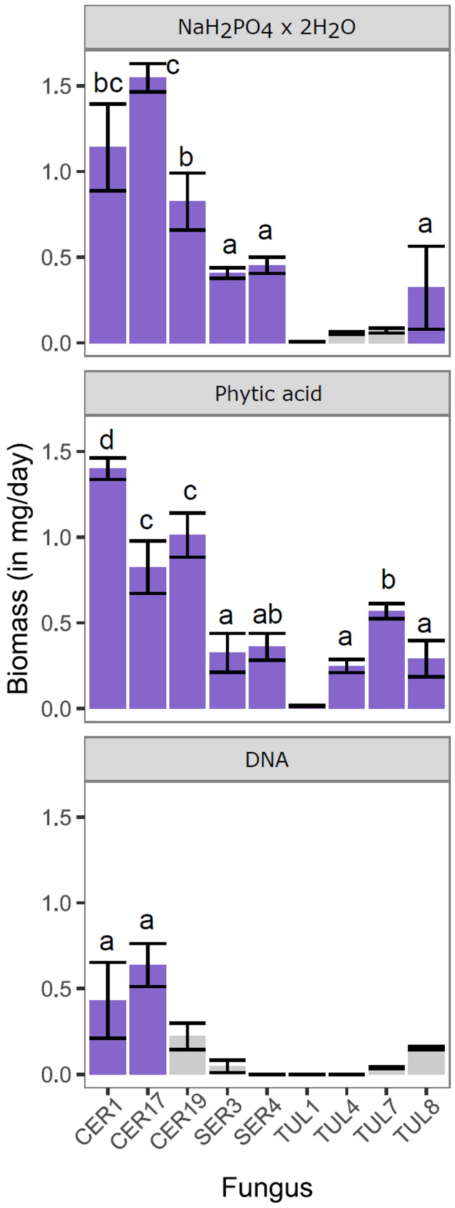

Figure S6. Mean biomass (in mg/day) ± SD (n=3 or 4) of the tested isolates (for which the biomass was significantly different from the control, in dark green) in liquid medium containing three different phosphorus sources. Different letters indicate a significant difference in the biomass increase ($P < 0.05$) between the different isolates and per each substrate. Grey bars indicate isolates with no significant biomass increase.

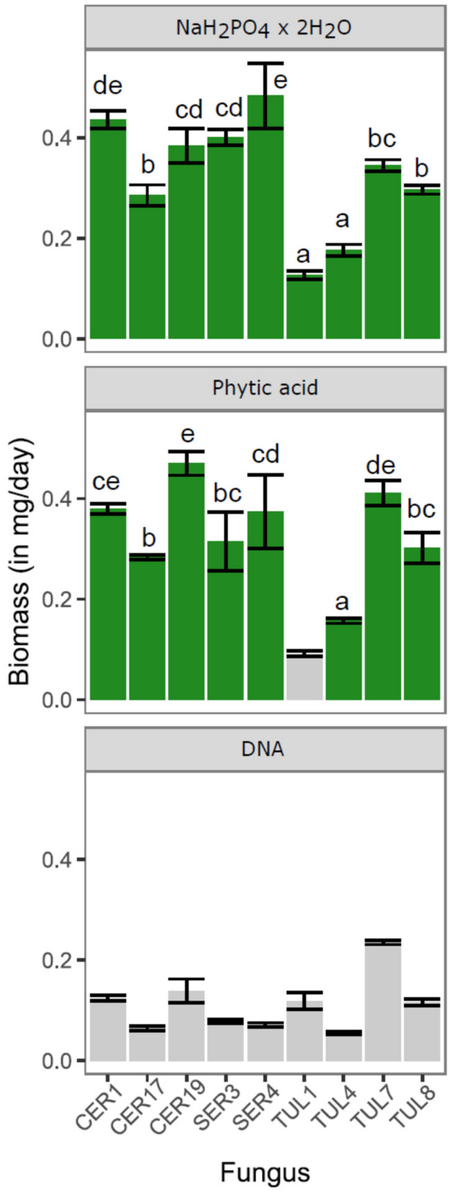

Supplement: Supplementary file 1 [file jof-09-00088-s001.zip › Supplementary figures.pdf]
